# The Proximal J Kappa Germline-Transcript Promoter Facilitates Receptor Editing through Control of Ordered Recombination

**DOI:** 10.1371/journal.pone.0113824

**Published:** 2015-01-05

**Authors:** Christian Vettermann, Greg A. Timblin, Vivian Lim, Ernest C. Lai, Mark S. Schlissel

**Affiliations:** 1 Division of Immunology & Pathogenesis, Department of Molecular & Cell Biology, University of California, Berkeley, California, United States of America; 2 Division of Molecular Immunology, Nikolaus Fiebiger Center for Molecular Medicine, University of Erlangen-Nürnberg, Erlangen, Germany; Tulane University, UNITED STATES

## Abstract

V(D)J recombination creates antibody light chain diversity by joining a Vκ gene segment with one of four Jκ segments. Two Jκ germline-transcript (GT) promoters control Vκ-Jκ joining, but the mechanisms that govern Jκ choice are unclear. Here, we show in gene-targeted mice that the proximal GT promoter helps targeting rearrangements to Jκ1 by preventing premature DNA breaks at Jκ2. Consequently, cells lacking the proximal GT promoter show a biased utilization of downstream Jκ segments, resulting in a diminished potential for receptor editing. Surprisingly, the proximal—in contrast to the distal—GT promoter is transcriptionally inactive prior to Igκ recombination, indicating that its role in Jκ choice is independent of classical promoter function. Removal of the proximal GT promoter increases H3K4me3 levels at Jκ segments, suggesting that this promoter could act as a suppressor of recombination by limiting chromatin accessibility to RAG. Our findings identify the first *cis*-element critical for Jκ choice and demonstrate that ordered Igκ recombination facilitates receptor editing.

## Introduction

Immunoglobulin (Ig) kappa (κ) light chain genes are generated from Vκ and Jκ gene segments during B cell development through a site-specific recombination process known as V(D)J recombination [1–3]. V(D)J recombination is initiated by the lymphocyte-specific RAG enzyme which cleaves DNA at conserved recombination signal sequences (RSSs) [4]. RAG cleavage is regulated by RSS substrate accessibility, a paradigm that has been termed the accessibility hypothesis [5,6].

One hallmark of accessible Igκ chromatin is Jκ germline transcription that coincides with the onset of recombination in pre-B cells [7,8]. Jκ germline transcription initiates at two different germline-transcript (GT) promoters, the distal and the proximal GT promoter, located 3.5 kilobases or about 50 basepairs, respectively, upstream of the 5’ most Jκ segment Jκ1 [8–10]. Transcriptional elongation from GT promoters likely plays a direct role in activating V(D)J recombination [11,12]. Consistent with this idea, deletion of a 4-kb region comprising both Jκ GT promoters substantially diminishes Igκ recombination [13]. Additionally, Igκ recombination is severely impaired in double knock-out mice lacking the Eiκ/3’Eκ or 3’Eκ/Edκ enhancers that stimulate GT promoters [14,15].

A unique feature of the Igκ locus is its ability to undergo secondary rearrangements by joining a new Vκ segment with a Jκ segment located downstream of an existing VκJκ exon [16]. Secondary Igκ rearrangements form the basis for receptor editing, a process by which immature B cells replace their Igκ chain in an attempt to generate a functional, non-autoreactive BCR [17–20]. Based on the delayed activation of Igλ genes, it has been estimated that each Igκ allele has enough time to undergo up to three rearrangements [21], which corresponds roughly to the total number of functional Jκ segments (Only Jκ1, Jκ2, Jκ4 and Jκ5 are functional gene segments; Jκ3 has a mutated RSS and thus is not recognized by RAG). A prerequisite for secondary Igκ rearrangements is the availability of Jκ segments located 3’ of an existing VκJκ exon. In other words, receptor editing should be most efficient if primary rearrangements target the 5’ end of the Jκ region.

Interestingly, while Vκ segments are selected for recombination from an array of about 140 segments without any pre-determined spatial order [22], most primary Vκ-Jκ rearrangements indeed target the 5’ most Jκ segment Jκ1 [23]. The other three segments Jκ2, Jκ4 and Jκ5 have a three- to seven-fold lower probability of being utilized in the first recombination attempt [23]. However, the mechanisms that govern the internal order of Igκ recombination and establish this bias in Jκ choice are poorly understood.

Given its proximity to the Jκ1 segment, we aimed to elucidate whether the proximal Jκ GT promoter plays a role in regulating Jκ choice. Gene-targeting in mice demonstrated that this promoter helps targeting primary rearrangements to Jκ1 by preventing premature DNA breaks at Jκ2. This in turn facilitates efficient receptor editing of the Igκ locus. We also provide evidence that the proximal GT promoter could serve as a suppressor of recombination by limiting H3K4me3 levels and transcription in the Jκ region.

## Results

### The proximal GT promoter influences ordered Igκ recombination

To determine the role of the proximal GT promoter in Igκ recombination, we deleted this promoter by gene-targeting in mice. A simple deletion, however, would bring the distal GT promoter much closer to the Jκ region, potentially leading to unpredictable secondary effects. To circumvent this problem, we first replaced the proximal GT promoter with a frt-flanked stuffer sequence of the same length and called the resulting allele κS (SI 1). The stuffer was then removed by crossing mice carrying the κS allele with mice expressing Flp recombinase in the germline (Actin-Flp), resulting in a pure deletion of the proximal GT promoter in the so-called κD allele. Using this strategy, we could infer that any phenotype observed with both κS and κD alleles was caused by the removal of the proximal GT promoter as opposed to changes in the spatial structure of the Igκ locus.

**Figure 1:**
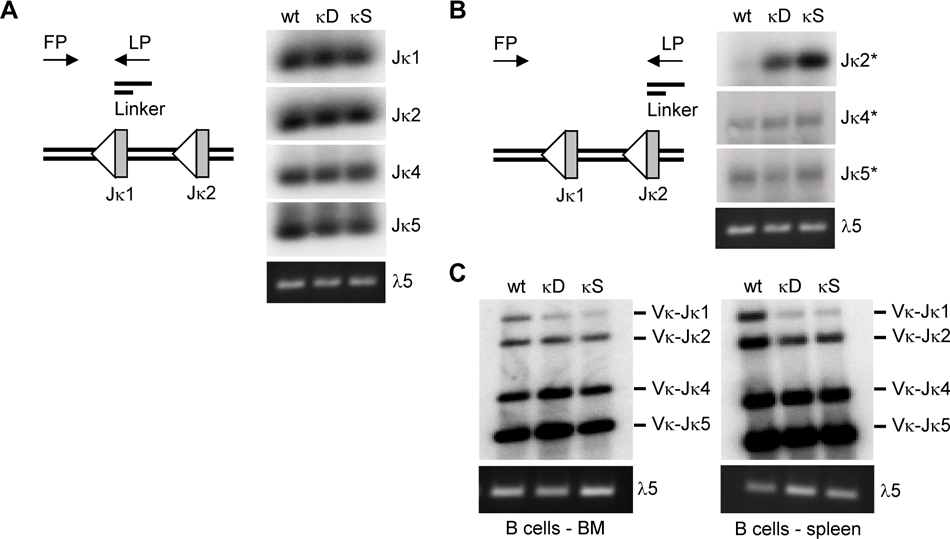
The proximal Jκ GT promoter controls Jκ choice. **A)** LM-PCR detects total DNA breaks at Jκ gene segments in pre-B cells from wildtype mice (wt) or mice lacking the proximal GT promoter (κD, deletion; κS, stuffer). Linker ligated genomic DNA was first amplified with several Jκ-specific forward primers (FP) and a linker-specific reverse primer (LP) and then hybridized with Jκ RSS probes. Results are representative of at least two independent experiments. **B)** LM-PCR detects premature DNA breaks at Jκ2, Jκ4, and Jκ5 in pre-B cells from wildtype mice (wt) or mice lacking the proximal GT promoter (κD, deletion; κS, stuffer). Linker ligated genomic DNA was first amplified with several Jκ-specific forward primers (FP) and a linker-specific reverse primer (LP) and then hybridized with Jκ RSS probes. Results are representative of at least two independent experiments. **C)** VJ coding joint PCR detects individual Jκ segments in completed VκJκ joints in B cells from bone marrow or spleen of wildtype mice (wt) or mice lacking the proximal GT promoter (κD, deletion; κS, stuffer). Genomic DNA was first amplified with a degenerate Vκ-specific forward primer and a reverse primer (MAR35) that binds downstream of Jκ5 and then hybridized with a probe (5’MAR35) that binds downstream of Jκ5 but upstream of the reverse primer. Results are representative of at least two independent experiments.

To determine whether the proximal GT promoter has an impact on the total level of Igκ recombination, we used LM-PCR to measure double-stranded DNA breaks that occur at recombination signal sequences (RSSs) upstream of each Jκ segment (1A, left). The overall abundance of DNA breaks in the Jκ region in pre-B cells was unchanged in the presence (wt) or absence (κD, κS) of the proximal GT promoter, demonstrating that the remaining distal GT promoter is sufficient to activate Igκ recombination (Fig. 1A, right).

LM-PCR can also detect premature (out-of-order) DNA breaks, for example those that occur at the Jκ2 segment when the Jκ1 segment has not been rearranged yet (Fig. 1B, left). Using appropriate primers, we found a sharp increase in premature Jκ2 breaks in pre-B cells lacking the proximal GT promoter (κD, κS), while premature Jκ4 and Jκ5 breaks remained unaffected in these cells, demonstrating that this promoter controls Jκ choice (Fig. 1B, right).

Premature DNA breaks at Jκ2 can lead to VκJκ2 joints, thereby skipping an available Jκ1 segment. This should diminish the utilization of Jκ1 in completed VκJκ joints. To test this prediction, we analyzed VκJκ joints in B cells from bone marrow or spleen of mice lacking the proximal GT promoter (κD, κS) and found an under-representation of Jκ1, thus implying Jκ1 skipping in favor of downstream Jκ segments (Fig. 1C). These findings suggest that the proximal GT promoter maintains the internal order of Jκ rearrangments to establish a balanced antibody light chain repertoire.

### The proximal GT promoter is inactive prior to Igκ recombination

Next we examined whether the role of the proximal GT promoter in Jκ choice can be linked to its transcriptional activation in pre-B cells. We took advantage of two previously generated Igκ reporter alleles [24,25]: one contains a GFP coding region inserted into the Jκ1 segment, which reports from the proximal GT promoter (κGFP), while the other contains a truncated hCD4 coding region under the control of the distal GT promoter (κhCD4) and served as a positive control.

**Figure 2:**
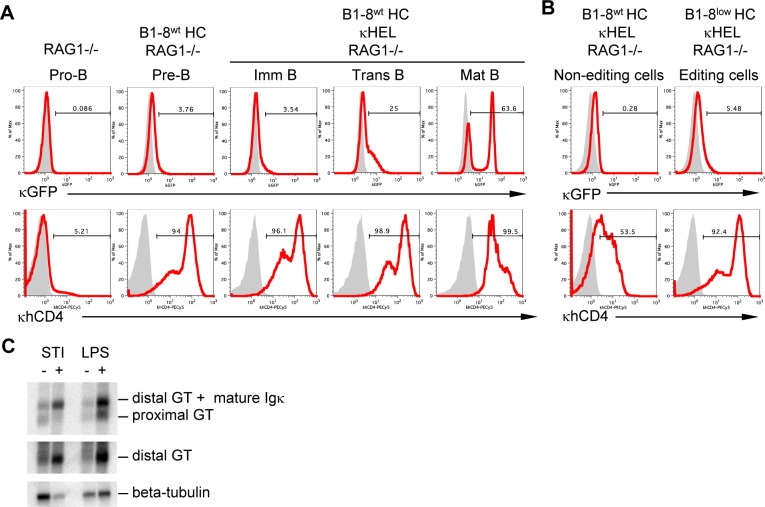
The proximal Jκ GT promoter is inactive prior to Igκ recombination. **A)** Flow cytometry detects expression of κGFP (proximal GT promoter reporter; top) and κhCD4 (distal GT promoter reporter; bottom) in RAG-deficient developing B cells carrying either no transgene, a B1-8^wt^ HC transgene, or a B1-8^wt^ HC transgene plus a κHEL transgene. Pro-B and pre-B cells are gated B220^+^ IgM^−^, immature (imm) B cells are gated B220^+^ IgM^+^ IgD^−^, transitional (trans) B cells are gated B220^+^ IgM^+^ IgD^low^, and mature (mat) B cells are gated B220^+^ IgM^+^ IgD^high^. Grey shaded histograms show cells from a C57Bl/6 control mouse. Results are representative of at least two independent experiments. **B)** Flow cytometry detects expression of κGFP (top) and κhCD4 (bottom) in non-editing (B1-8^wt^HC/κHEL/RAG−/−) or receptor-editing (B1-8^low^HC/κHEL/RAG−/−) B cells (gated B220+ IgM−). Grey shaded histograms show cells from a C57Bl/6 control mouse. Results are representative of at least two independent experiments. **C)** Northern blotting of Jκ GTs in an Abelson virus-transformed pre-B cell line treated with either STI-571 (STI, which mimics pre-BCR signaling) or the TLR4 ligand LPS. mRNA was hybridized with a Cκ-specific probe (top) that recognizes mature Igκ transcripts, distal GTs, and proximal GTs, the latter of which can be identified by their smaller size. Additionally, the blot was hybridized with a probe specific for distal GTs (middle). Beta-tubulin transcripts (bottom) served as a loading control. Results are representative of two independent experiments.

To distinguish the activation of GT promoters prior to Igκ recombination in pre-B cells from promoter activation later in B cell development, we crossed each reporter allele onto a RAG1-deficient background. To mimic pre-BCR signals, we then crossed a pre-rearranged heavy chain transgene (B1-8^wt^) onto each RAG1-deficient Igκ reporter background and observed a substantial up-regulation of κhCD4 but not κGFP expression in pre-B cells (Fig. 2A, left). This demonstrates that only the distal but not the proximal GT promoter is fully active prior to Igκ recombination. The lack of GFP expression in pre-B cells was not caused by a defective reporter allele, since mature B cells with a complete BCR (RAG1^−/−^/B1-8^wt^/κHEL) were able to express GFP from the proximal GT promoter (Fig. 2A, right).

Igκ recombination continues in cells undergoing receptor editing. To determine which GT promoter is activated during receptor editing, we analyzed κGFP and κhCD4 expression in RAG-deficient B cells with either an innocuous (B1-8^wt^/κHEL) or autoreactive (B1-8^low^/κHEL) BCR. Autoreactive BCR signals upregulated κhCD4 but not κGFP expression, demonstrating that only the distal but not the proximal GT promoter is strongly active in receptor-editing B cells (Fig. 2B).

To ensure that Igκ reporter alleles adequately reflect the underlying biological regulation, we also analyzed distal and proximal GT promoter transcripts from the unmodified Igκ locus, using an Abelson virus transformed pre-B cell line. Pre-BCR signaling can be mimicked in these cells by treatment with the Abelson kinase inhibitor STI-571 [26]. Northern blot analysis showed that cells treated with STI-571 strongly upregulated distal but not proximal GT promoter transcripts (Fig. 2C, left lanes). As a positive control, we treated cells with the toll-like receptor 4 (TLR4) ligand LPS, which is known to activate both GT promoters (S2 Fig.), a fact that we confirmed in our Northern blot experiment (Fig. 2C, right lanes).

Together, these results show that neither primary Igκ recombination nor receptor editing correlate with the transcriptional activation of the proximal GT promoter, indicating that the role of this promoter in Jκ choice is independent of classical promoter function.

### The proximal GT promoter limits H3K4me3 levels and transcription in the Jκ region

**Figure 3:**
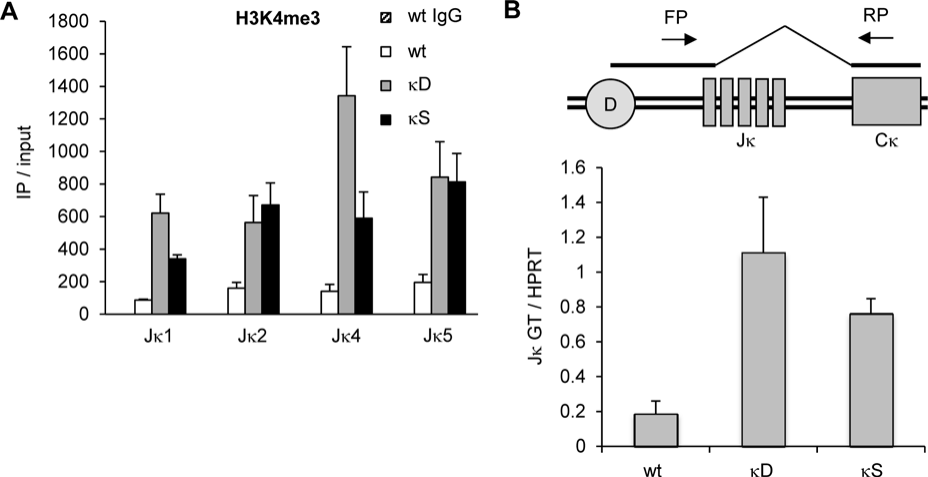
Removal of the proximal Jκ GT promoter increases H3K4me3 levels in the Jκ region and upregulates distal GT promoter activity. **A)** ChIP analysis of H3K4me3 levels in pre-B cells from wildtype mice (wt) or mice lacking the proximal GT promoter (κD, deletion; κS, stuffer). Immunoprecipitated genomic DNA was analyzed by qPCR. Specific enrichment was calculated with the formula 2^Ct(Input)-Ct(IP)^. Results are representative of two independent experiments. Error bars represent standard deviations of triplicate qPCR assays. **B)** RT-qPCR analysis of distal GT promoter activity in pre-B cells from wildtype mice (wt) or mice lacking the proximal GT promoter (κD, deletion; κS, stuffer). Jκ GT specific amplification was normalized to HPRT. Locations of forward (FP) and reverse (RP) primers are indicated above the diagram (D, distal GT promoter). Results are representative of two independent experiments. Error bars represent standard deviations of triplicate qPCR assays.

The chromatin mark H3K4me3 was previously shown to recruit RAG and enhance DNA cleavage at RSSs [27–29]. We therefore hypothesized that premature Jκ2 breaks in the absence of the proximal GT promoter resulted from higher H3K4me3 levels. Chromatin immunoprecipitation (ChIP) combined with qPCR revealed an increase of H3K4me3 in the Jκ region of pre-B cells lacking the proximal GT promoter (Fig. 3A), suggesting that this promoter could act as a local suppressor of recombination by restricting RSS accessibility to RAG. In this experiment, qPCR primers for Jκ1 selectively detected H3K4me3 on non-rearranged Igκ alleles, while qPCR primers for Jκ2, Jκ4, and Jκ5 did not distinguish between rearranged and non-rearranged alleles. Despite this ambiguity, the modulation of H3K4me3 levels likely occurs on non-rearranged Igκ alleles, because the abundance of Jκ2-, Jκ4-, and Jκ5-rearranged alleles in mutant and wildtype mice is very similar (Fig. 1C). Therefore, the presence of these rearranged Igκ alleles would not introduce a bias into H3K4me3 measurements. Additionally, the proximal GT promoter is not present (or has been moved far away from the Jκ region) on rearranged Igκ alleles in both mutant and wildtype mice, and thus the increase in H3K4me3 levels in mutant mice most likely resulted from changes on non-rearranged Igκ alleles.

Since H3K4me3 marks active promoters in other loci and is highly correlated with transcription rates [30,31], we postulated that the increase in H3K4me3 in the Jκ region in pre-B cells lacking the proximal GT promoter resulted from a higher distal GT promoter activity. We therefore measured transcript levels with RT-qPCR and found more Jκ germline transcripts in pre-B cells from κD and κS mice compared to wildtype mice, suggesting that the distal GT promoter becomes more active when the proximal GT promoter has been removed (Fig. 3B). Alternatively, increased levels of Jκ germline transcripts in mutant pre-B cells could have resulted from a higher percentage of non-rearranged Igκ alleles, which is a known consequence of impaired Igκ recombination and delayed developmental progression of pre-B cells. However, we found no evidence of abnormal B cell development in mutant mice (S3 Fig.) and the proportion of rearranging Igκ alleles was comparable between mutant and wildtype pre-B cells (Fig. 1A, “total Jκ breaks”). Hence, it is fair to assume that the percentage of non-rearranged Igκ alleles was similar for all pre-B cells used in this experiment.

Together, these results show that the proximal GT promoter restricts both H3K4me3 chromatin modifications and transcription in the Jκ region, both of which are key regulators of RAG recruitment and RSS accessibility. Thus, in mutant mice lacking the proximal GT promoter, the RAG complex is more likely to target downstream Jκ RSSs for premature DNA cleavage (see Discussion for why Jκ2 may be preferentially targeted over Jκ4 and Jκ5).

### The removal of the proximal GT promoter diminishes the potential for receptor editing in the Igκ locus

Most primary VκJκ joints in wildtype pre-B cells are made to the Jκ1 segment [23], leaving three downstream segments (Jκ2, Jκ4, and Jκ5) available for receptor editing. In the absence of the proximal GT promoter, however, primary rearrangements more frequently skip the Jκ1 segment and prematurely target Jκ2, which leaves only two possibilities to replace an autoreactive VκJκ exon through secondary rearrangements with Jκ4 and Jκ5. Therefore, premature Jκ2 rearrangements are predicted to reduce the potential for receptor editing in the Igκ locus.

**Figure 4:**
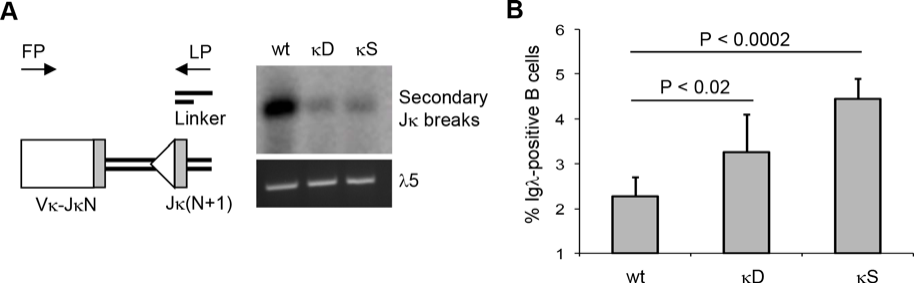
Reduced potential for Igκ editing and increased number of Igλ-positive B cells in mice lacking the proximal Jκ GT promoter. **A)** LM-PCR detects secondary DNA breaks indicative of receptor editing in pre-B cells from wildtype mice (wt) or mice lacking the proximal GT promoter (κD, deletion; κS, stuffer). Linker ligated genomic DNA was first amplified with a degenerate Vκ forward primer (FP) and a linker-specific reverse primer (LP) and then hybridized with Jκ RSS probes. Results are representative of two independent experiments. **B)** Flow cytometry detects Igλ expression in B220^+^ splenic B cells from wildtype mice (wt) or mice lacking the proximal GT promoter (κD, deletion; κS, stuffer). The bar diagram shows the mean values for the percentage of Igλ-positive cells from at least three independent experiments for each genotype. Error bars represent standard deviations, P values were calculated with a Student’s t-test.

To test this prediction, we analyzed secondary DNA breaks that occur at Jκ segments after a primary VκJκ joint has been formed, thus indicating continuous RAG activity and receptor editing. Secondary DNA breaks at Jκ segments can be detected by LM-PCR using a universal degenerate Vκ forward primer and a linker-specific reverse primer (Fig. 4A, left). By comparing PCR products with the expected length for both secondary Jκ2 and Jκ5 breaks, we found a striking decrease in secondary DNA breaks in developing B cells from κD and κS mice, demonstrating a diminished potential for Igκ editing upon removal of the proximal GT promoter (Fig. 4A, right). Even though the assay was designed to detect both secondary Jκ2 and Jκ5 breaks, the decrease in secondary breaks is most likely to reflect reduced Jκ1 to Jκ2 editing, due to the fact that more Igκ alleles undergo their primary rearrangement to Jκ2 in mutant mice. Once the primary rearrangement has occurred, however, and the GT promoters have been removed, there is no obvious mechanism that would account for reduced Jκ4 to Jκ5 editing in mutant mice.

Since the distribution of cells across various stages of B cell development was normal in mice lacking the proximal GT promoter (S3 Fig.), we reasoned that developing B cells may compensate for diminished Igκ editing by more frequently switching to Igλ. Consistent with this hypothesis, mice lacking the proximal GT promoter showed higher numbers of Igλ-positive mature B cells (Fig. 4B), suggesting that increased switching to Igλ compensates for the exhaustion of editing possibilities in the Igκ locus.

## Discussion

Transcription from the distal and proximal Jκ GT promoters is thought to induce an open chromatin structure that activates the Igκ locus and facilitates RAG cleavage. However, the relative contributions of the two GT promoters to Igκ activation are controversial [24,25,32,33]. Our study establishes that the distal GT promoter is sufficient to activate the Jκ region for recombination, even in the absence of the proximal GT promoter (Fig. 1A). This finding is in accordance with a previous report showing that most Jκ GTs in a population of pre-B cells start at the distal promoter [24]. Yet it remained unclear whether all pre-B cells expressed only minimal levels of proximal GTs or whether high proximal GT promoter activity was restricted to only a small fraction of pre-B cells [25,32]. Our experiments using κGFP reporter mice crossed onto a RAG^−/−^/B1-8^wt^ background answered this question by demonstrating that the proximal GT promoter is essentially inactive in all pre-B cells prior to Igκ recombination (Fig. 2A).

One possible explanation for this observation could be promoter interference, by which the upstream activation of the distal GT promoter hinders the downstream activation of the proximal one. In accordance, the highest proximal GT promoter activity is found in mature B cells that exhibit the lowest distal GT promoter activity (Fig. 2A). Promoter interference was observed in the structurally related TCRα locus, where the T early alpha promoter suppresses the activation of downstream GT promoters in the Jα region [12,34]. Future studies in mice lacking the distal Jκ GT promoter could address whether this phenomenon also occurs in the Igκ locus.

It is currently unclear why the proximal GT promoter is activated in over 50% of mature B cells in the bone marrow (Fig. 2A), i.e., at a time when Igκ recombination has been successfully completed. There is controversy as to whether mature B cells attempt to undergo V(D)J recombination; but even if this occurred, it would be an extremely rare phenomenon at the Igκ locus (~1% of stimulated B cells, [35]), which would not fully explain the broad and constitutive activation of the proximal GT promoter. Interestingly, in the human system, Jκ GTs may not be sterile and encode a truncated protein designated JCκ [36]. When we tested this possibility in mice, however, we found that germline Igκ genes did not express JCκ protein at any stage during B cell development (S4 Fig.). We therefore postulate that the activation of the proximal GT promoter on a non-rearranged Igκ allele in mature B cells is simply a bystander effect of genetic regulations that are necessary to increase the expression of the rearranged Igκ allele.

The major finding of our study is that the proximal GT promoter plays a critical role in Jκ choice, despite its transcriptional inactivity in pre-B cells. Thus, the proximal GT promoter is the first *cis*-element known to regulate the internal order of Jκ rearrangements. In mice lacking the proximal GT promoter, we found a sharp increase in initial (premature) Jκ2 breaks (“Jκ1 skipping”, Fig. 1B), resulting in less frequent formation of VκJκ1 joints (Fig. 1C). Surprisingly, this was not accompanied by a concomitant decrease in total Jκ1 breaks (Fig. 1A). One possible explanation could be that a Jκ1 break can still occur *after* an initial Jκ2 break, but since this Jκ1 break would be located on an extrachromosomal circle, it could not form a VκJκ1 joint. Similarly, it may be somewhat puzzling at first glance why elevated levels of premature Jκ2 breaks in mice lacking the proximal GT promoter (Fig. 1B) did not result in higher levels of total Jκ2 breaks (Fig. 1A). The most plausible explanation is that the fraction of premature Jκ2 breaks amongst all Jκ2 breaks could still be relatively small, e.g. 20%, in which case the increase in total Jκ2 breaks (~1.2-fold) would likely be below the detection limit of our assay.

Previously, the utilization of individual Ig gene segments during rearrangement was thought to be mainly controlled by recombination efficiencies of individual RSSs [37,38]. Recombination efficiencies are determined by RSS sequence variations [22,39] and can be predicted with great accuracy using an algorithm that calculates recombination information content (RIC) scores [40,41]. RIC scores are logarithmic values that range from 0 to −1000, with 0 representing the highest recombination efficiency. The RIC scores for Jκ RSSs are as follows: Jκ1: −27, Jκ2: −30, Jκ4: −36, and Jκ5: −35 [42]. These scores are consistent with the biased utilization of Jκ segments in primary rearrangements [23]. How could the proximal GT promoter cooperate with this layer of regulation?

Our results suggest that the proximal GT promoter limits RAG cleavage by keeping H3K4me3 levels in the Jκ region below a certain threshold (Fig. 3A). Interestingly, the high intrinsic recombination efficiency of the Jκ1 RSS, reflected in its high RIC score, could allow for maximal RAG cleavage even at these lower H3K4me3 levels [37]. However, downstream RSSs, such as the Jκ2 RSS, that have lower RIC scores likely require additional activation for RAG binding and cleavage, and could therefore be far more sensitive to a fine-tuned modulation of H3K4me3 levels mediated by the proximal GT promoter. Consequently, when the proximal GT promoter is removed by gene-targeting, the resulting higher H3K4me3 levels could allow RAG to prematurely cleave the Jκ2 RSS (Fig. 1B). In contrast, Jκ4 and Jκ5 RSSs are not cleaved prematurely in the absence of the proximal GT promoter, because they have even lower RIC scores than the Jκ2 RSS. Interestingly, under physiological conditions, primary rearrangements to Jκ1 delete the proximal GT promoter (or move it very far away from the Jκ region in the case of an inversion), and thus terminate its suppressive effects on downstream Jκ segments. This could therefore help to generate DNA breaks at Jκ2 only after the Jκ1 segment has been utilized.

How does the proximal GT promoter keep H3K4me3 levels in check? One mechanism could be its transcriptional inactivity in pre-B cells: The highest H3K4me3 levels are typically found within a 2-kb region upstream and downstream of transcription start sites (TSS) [30]. Since the TSS of the proximal GT promoter is located within 50 basepairs upstream of the Jκ region, it appears likely that high promoter activity would induce massive H3K4me3 deposition, in particular at Jκ1 and Jκ2. This could be part of the reason why H3K4me3 levels are increased in mice carrying a deletion of the proximal GT promoter (κD), since the strongly active distal GT promoter is much closer to the Jκ region in these mice.

However, since H3K4me3 levels were also increased in κS mice, in which a stuffer region keeps the distal GT promoter at its regular distance, there must be an additional mechanism. We show here that distal GT promoter activity is up-regulated in the absence of the proximal GT promoter (Fig. 3B), suggesting that there is an inhibitory relationship between these two promoters. One possibility could be that the proximal GT promoter constitutes a roadblock for traveling RNA polymerase II that started at the distal promoter. The roadblock may consist of transcription factors such as Pax5 that binds to the KII/KI sites upstream of Jκ1 [43]. Accordingly, dissociation of Pax5 from the KII/KI sites was shown to correlate with the induction of Igκ recombination [44]. Another roadblock could be paused RNA polymerase II that may be stalled at the proximal GT promoter, similar to what has been observed for Vκ promoters [45]. Alternatively, the proximal GT promoter could compete with the distal GT promoter for access to Igκ enhancers by forming inhibitory chromatin anti-loops [46]. All of these mechanisms could potentially explain how the proximal GT promoter reduces distal GT promoter activity and thus limits the abundance of H3K4me3 in the Jκ region.

Finally, our study provides the first empirical evidence that the regulation of Jκ choice affects the process of receptor editing. When primary rearrangements are less biased toward Jκ1, as is the case in the absence of the proximal GT promoter, developing B cells have fewer chances for undergoing Igκ editing (Fig. 4A). This is likely caused by the diminished number of available downstream Jκ segments resulting from Jκ1 skipping due to premature Jκ2 breaks, which illustrates the importance of the non-stochastic 5’-3’ order of Jκ rearrangements. A reduced potential for receptor editing in the Igκ locus could be detrimental to developing B cells in cases where antibody heavy chains cannot pair with Igλ chains or would form autoreactive Igλ-BCRs. Thus, multiple chances to edit the Igκ gene could be key for establishing a diverse and non-autoreactive antibody repertoire.

## Materials and Methods

### Ethics Statement

All animal experiments in this study were approved by the Animal Care and Use Committee of the University of California, Berkeley (Permit number: MAUP R253) and conducted in accordance with the recommendations in the Guide for the Care and Use of Laboratory Animals of the National Institutes of Health.

### Mice

κD and κS mice were generated by gene-targeting in Prm1-Cre 129Sv ES cells [25], as described below (under Southern Blots and genotyping PCRs for κD and κS alleles) and in S1 Fig., and carry a deletion of the proximal κGT promoter (κD) or a stuffer sequence (from an intergenic region of the human genome) in place of the proximal κGT promoter (κS). κGFP reporter mice contain a GFP cDNA, an SV40 intron, and a SV40 polyA site, which were inserted into the first Jκ gene segment, i.e. downstream of the proximal κGT promoter [25]. κhCD4 reporter mice contain a human CD4 (hCD4) cDNA, an SV40 intron, and a SV40 polyA site, which were inserted downstream of the distal κGT promoter [24]. B1-8^wt^/κHEL/RAG1^−/−^ mice carry pre-rearranged IgH (B1-8^wt^) and IgL (κHEL) genes that give rise to a non-autoreactive BCR, and B1-8^low^/κHEL/RAG1^−/−^ mice carry pre-rearranged IgH (B1-8^low^) and IgL (κHEL) genes that give rise to an autoreactive BCR [47]. Each of these two strains was crossed into a RAG1-deficient background [48]. hCκ mice carry a human Cκ segment in place of the murine Cκ segment [47].

### Southern blots and genotyping PCRs for κD and κS alleles

*BamHI*-digested genomic DNA was analyzed by Southern blotting with probes labeled with ^32^P-dCTP (Perkin Elmer) using the RadPrime kit (Invitrogen). 5’probe: amplified from genomic DNA with primers ATATCTATCATCCTGCACAAAAATCAATTC and CCCCAACCCTGCCGCTACTCTGTGTAGCC; J probe: amplified from genomic DNA with primers GAGGGTTTTTGTACAGCCAGACAGTGG and TCAATAACTACTCATGCTTATTCTCCG. Radioactive bands were visualized with a Typhoon Trio phosphoimager (Amersham) and analyzed with ImageQuant 5.2 software (GE). Genotyping PCRs were performed using mouse tail DNA (κD mice: cv248 TGGCTGTAGCCTAATGTCCTTCTG, cv249 GAATATTCTTGTCTGAGAGCTGCC, cv250: TGCCAGAATCTGGTTTCAGAGTAAG; κS mice: cv248, cv249, cv251: CAGCTACTAAAGCGAAACAAGCATC). PCR conditions: 94°C, 3 min; 35x (94°C, 20 s / 60°C, 1 min / 72°C, 1 min); 72°C, 7 min.

### Cells

Primary B lineage cells were isolated from bone marrow and spleen by using anti-CD19 magnetic beads (Miltenyi) and cell sorting on MoFlow with anti-B220-PECy5 and anti-IgM-FITC antibodies. Pre-B cells were purified as B220^low^ IgM− cells, B cells were purified as B220+ IgM+ cells. Cells were cultured in RPMI 1640 (supplemented with 5% FCS, 2 mM L-glutamine, 50 mM β-ME, 100 U/mL penicillin, and 100 μg/mL streptomycin) and grown at 37°C in 5% CO_2_. 10 μg/mL LPS (L6529, Sigma), 2 μM CpG ODN 1826 (InvivoGen), or 2.5 μM STI-571 (Gleevec, Novartis) were added as indicated.

### LM-PCR and VJ coding joint PCR

Genomic DNA for LM-PCR and VJ coding joint PCR assays was purified by using proteinase K digest and phenol/chloroform extraction. For LM-PCR analysis, two oligos (BW-1: GCGGTGACCCGGGAGATCTGAATTC, BW-2: GAATTCAGATC) were annealed to create the double-stranded DNA linker that was ligated to genomic DNA breaks with T4 DNA ligase. PCR was perfomed using a linker primer (BW-H CCGGGAGATCTGAATTCCAC) and either a Jκ-specific forward primer to detect primary DNA breaks (Jκ1-cv9: AGCTTTCGCAGCTACCCACTGCTCTGT, Jκ2-ko5: CAGAAATGCTCAAAGAAGCAGGGTAGCCTG, Jκ4-ko5: TACTGTACAAGCTGAGCAAACAGACTGACCTC, Jκ5-ko5: GTAAGGGGAATGTAGAAGAAAGAGCTGGGC) or a degenerate Vκ primer to detect secondary DNA breaks (VκS: CCGAATTCGSTTCAGTGGCAGTGGRTCRGGRAC). Touch-down PCR conditions: 94°C, 1 min; 11× (92°C, 30 s / 70–60°C, 2.5 min, with ΔT = 1°C/cycle); 25× (92°C, 30 s / 60°C, 2.5 min); 72°C, 10 min. PCR products were hybridized in 6x SSPE / 5× Denhardt’s / 0.1% SDS with a mix of four different Jκ RSS probes labelled with γ^32^P-ATP (Perkin Elmer) using the RadPrime kit (Invitrogen). Jκ1-RSS probe (ko6): AGCCAGACAGTGGAGTACTACCAC, Jκ2-RSS probe: TGGGGGTTGAGTGAAGGGACACCAG, Jκ4-RSS probe: AAGGGGGGCGCAGTGATATGAATCAC, Jκ5-RSS probe: GAGAGGGGCATGTCATAGTCCTCAC. Premature DNA breaks were discriminated from regular DNA breaks in the Jκ region based on the different size of the PCR products.

VJ-PCR was performed on genomic DNA with a degenerate Vκ primer (VκD: GGCTGCAGSTTCAGTGGCAGTGGRTCWGGRAC) and a primer that anneals downstream of Jκ5 (MAR35: AACACTGGATAAAGCAGTTTATGCCCTTTC). PCR conditions: 95°C, 3 min; 25× (95°C, 20s / 60°C, 1 min / 72°C, 4 min); 72°C, 8 min. PCR products were in hybridized with a probe labelled with γ^32^P-ATP using the RadPrime kit (Invitrogen). 5’MAR35 probe: GAGAACAGAGATGTGACAGACTACACTAATGTGAG. In all assays, blots were prehybridzed for an hour and then hybridized with the probes overnight in 6× SSPE / 5× Denhardt’s / 0.1% SDS at 56°C. Blots were washed in 2x SSC / 0.1% SDS at 42°C for 30 min. A PCR detecting the λ5 gene (TW40: TCCCCAGGCAGTGTGAAGTTCTCC, TW41:GGCCTTGCAATTGATCGAGGTACC) served as a loading control.

### Flow cytometry

Flow cytometry was performed with the following antibodies: anti-B220-PE (clone RA3-6B2, eBioscience), anti-B220-PECy5 (clone RA3-6B2, BD), anti-B220-PETexasRed (clone RA3-6B2, Invitrogen), anti-IgM-FITC (clone II/41, eBioscience), anti-IgM-PECy5 (clone II/41, eBioscience), anti-IgD-PE (clone 11–26, eBioscience), anti-CD43-biotin (clone S7, BD), Streptavidin-PECy7 (cat. no. 557598, BD), anti-hCD4-PECy5 (clone RPA-T4, eBioscience), anti-Igκ-PE (clone 187.1, BD), anti-Igλ1,2,3-FITC (clone R26-46, BD), anti-human-Igκ-biotin (Fab)_2_ (cat. no. 2063–08, Southern). If indicated, Fix & Perm kit (Life Technologies / An der Grub Bio Research) was used for intracellular staining.

### Northern blots and RT-qPCR

For Northern blots, mRNA was purified using Oligotex Direct mRNA kit (Qiagen) and analyzed using NorthernMax kit (Ambion). 1–4 μg mRNA was loaded per lane and hybridized with α^32^P-dCTP (Perkin Elmer) probes labeled with RadPrime Kit (Invitrogen). Cκ probe: amplified from genomic DNA with primers GGGCTGATGCTGCACCAACTGTATCC and GACTGCCATGTAGTGGACAGCCAACC; distal κGT probe (located in the first exon of distal κGT promoter): amplified from genomic DNA with primers CCCTGATCCACTTACTGTTTTC and CTGGCACTGGAATATTCTTGTCTG. For RT-qPCR, RNA was isolated with Trizol LS, treated with Dnase I (Promega), reverse-transcribed with MMLV-RT (Invitrogen) using random hexamers, and analyzed by qPCR. Primers: forward: AGCTTTCGCAGCTACCCACTGCTCTGT; reverse: GGAAGATGGATACAGTTGGT.

### H3K4me3 ChIP

ChIP was performed as described [30]. Briefly, samples were crosslinked with formaldehyde, lysed, and sonicated for 20 seconds at constant 30% amplitude, with 40 seconds rest on ice for 22–30 cycles. Chromatin was precipitated 15 μg of H3K4me3-specific antibodies (ab8580, Abcam) coupled to Dynal beads and analyzed by qPCR. Primers: Jκ1 for (cv9: AGCTTTCGCAGCTACCCACTGCTCTGT), Jκ1 rev (cv336: CCACCACAGTGGTAGTACTCCACTGTC), Jκ2 for (cv349: CAGAAATGCTCAAAGAAGCAGGGTA; Jκ2 rev (cv350: TCCGAACGTGTACACACACTGGT), Jκ4 for (cv355: TGTGACGTTTTGGTTCTGTTTGG), Jκ4 rev (cv356: TTTCGCTCAGCTTTCACACTGAC), Jκ5 for (cv359: TTGACGTGGCATACAGTGTCAGA), Jκ5 rev (cv360: CTGCAGTCAGACCCAGATCTCAA)

## Supporting Information

S1 Fig Generation of mice lacking the proximal Jκ GT promoter. **A)** Schematic depiction of wildtype and gene-targeted Igκ alleles. The positions of BamHI restrictions sites and Southern blot hybridization probes are indicated. The white box in the κS allele depicts the frt-flanked stuffer. D, distal Jκ GT promoter; P, proximal Jκ GT promoter; E, intronic enhancer.
**B)** Southern blotting of genomic DNA from gene-targeted ES cells using BamHI digest. The κS allele is about 1 kb larger in ES cells than depicted in A) due to the presence of a floxed Neo cassette that was removed later in the male germline of the κS founder mice by Prm1-Cre expression. ES cell clone 83 displayed correct recombination of left and right homology arms. Results are representative of two independent experiments.
**C)** Southern blotting of genomic DNA from thymus of heterozygous κD and κS mice using BamHI digest was done after the mice had been crossed with Act-Flp deleter mice. Results are representative of two independent experiments.
(PDF)

S2 Fig TLR signaling activates both the proximal and the distal Jκ GT promoter. Flow cytometry detects Igκ reporter gene expression in splenic B cells from κGFP mice (top panel), κGFP/B1-8^wt^HC/κHEL/RAG−/− mice (second panel), and κhCD4 mice (third panel). Splenic B cells were first sorted for GFP-negative or hCD4-low expressing cells and then treated with LPS or CpG-DNA for four days. Grey shaded histograms show untreated κhCD4 cells (third panel) or cells from a C57Bl/6 control mouse (all other panels). Results are representative of at least two independent experiments.
(PDF)

S3 Fig B cell development in mice lacking the proximal Jκ GT promoter. Stages of B cell development were analyzed by flow cytometry in the bone marrow from wildtype mice (wt) or mice lacking the proximal GT promoter (κD, κS). Results are representative of at least three independent experiments.
(PDF)

S4 Fig Germline Igκ genes do not encode JCκ protein in mice. hCκ mice were crossed with B1-8^wt^ HC/κHEL mice and back-crossed onto a RAG−/− background to obtain the depicted genotypes. Bone marrow cells were stained for surface markers and then fixed and permeabilized to analyze human Cκ expression by flow cytometry. Pro-B and pre-B cells are gated B220^+^ IgM^−^, immature (imm) B cells are gated B220^+^ IgM^+^ IgD^−^, transitional (trans) B cells are gated B220^+^ IgM^+^ IgD^low^, and mature (mat) B cells are gated B220^+^ IgM^+^ IgD^high^. Mature B cells from a regular hCκ mouse served as a positive control. Grey shaded histograms show cells from a hCκ-negative control mouse. Results are representative of at least two independent experiments.
(PDF)
