# Supplementary figures and images for: The Proximal J Kappa Germline-Transcript Promoter Facilitates Receptor Editing through Control of Ordered Recombination
Source: PLoS One. 2015 Jan 5;10(1):e0113824. doi: 10.1371/journal.pone.0113824 (PMC4283955; doi:10.1371/journal.pone.0113824)

Figure S1

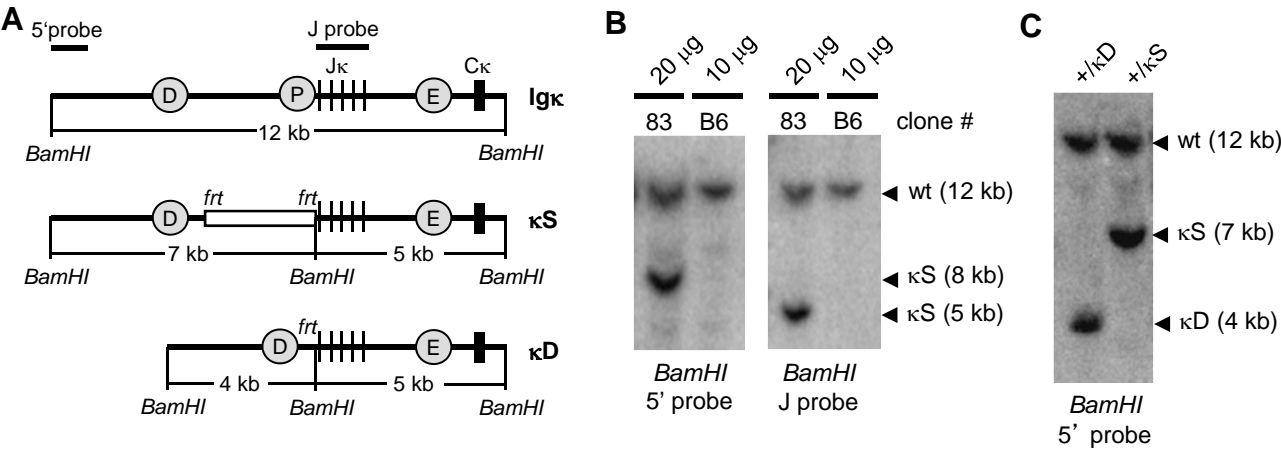

Supplement: S1 Fig — A) Schematic depiction of wildtype and gene-targeted Igκ alleles. The positions of BamHI restrictions sites and Southern blot hybridization probes are indicated. The white box in the κS allele depicts the frt-flanked stuffer. D, distal Jκ GT promoter; P, proximal Jκ GT promoter; E, intronic enhancer. B) Southern blotting of genomic DNA from gene-targeted ES cells using BamHI digest. The κS allele is about 1 kb larger in ES cells than depicted in A) due to the presence of a floxed Neo cassette that was removed later in the male germline of the κS founder mice by Prm1-Cre expression. ES cell clone 83 displayed correct recombination of left and right homology arms. Results are representative of two independent experiments. C) Southern blotting of genomic DNA from thymus of heterozygous κD and κS mice using BamHI digest was done after the mice had been crossed with Act-Flp deleter mice. Results are representative of two independent experiments. (PDF) [file pone.0113824.s001.pdf]

Figure S2

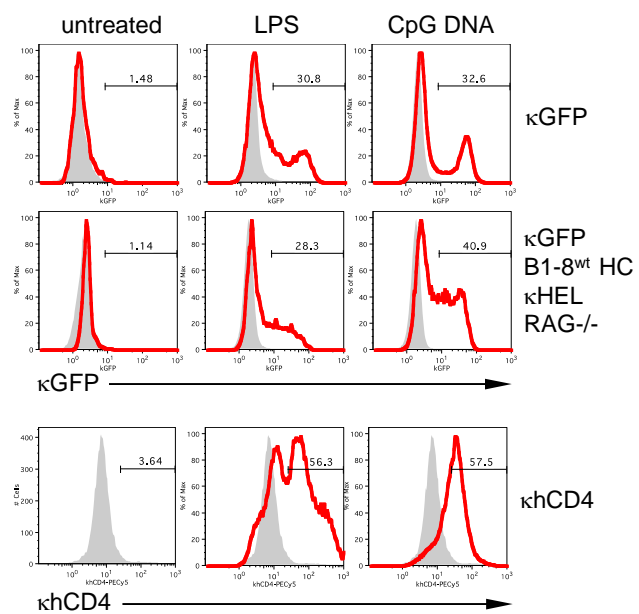

Supplement: S2 Fig — Flow cytometry detects Igκ reporter gene expression in splenic B cells from κGFP mice (top panel), κGFP/B1-8wtHC/κHEL/RAG−/− mice (second panel), and κhCD4 mice (third panel). Splenic B cells were first sorted for GFP-negative or hCD4-low expressing cells and then treated with LPS or CpG-DNA for four days. Grey shaded histograms show untreated κhCD4 cells (third panel) or cells from a C57Bl/6 control mouse (all other panels). Results are representative of at least two independent experiments. (PDF) [file pone.0113824.s002.pdf]

Figure S3

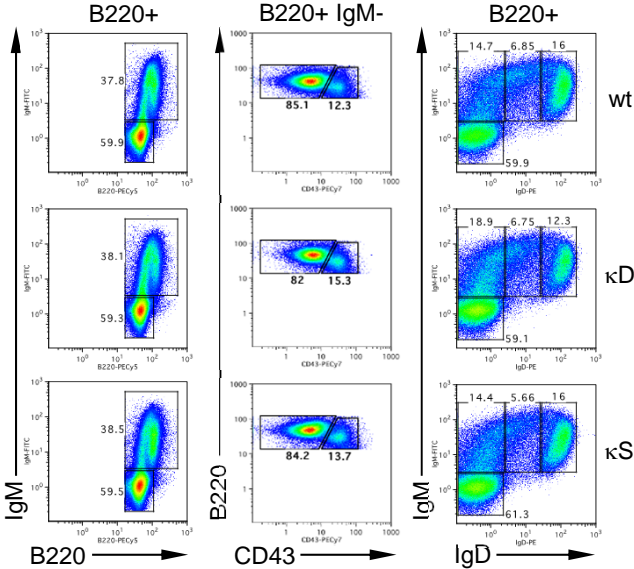

Supplement: S3 Fig — Stages of B cell development were analyzed by flow cytometry in the bone marrow from wildtype mice (wt) or mice lacking the proximal GT promoter (κD, κS). Results are representative of at least three independent experiments. (PDF) [file pone.0113824.s003.pdf]

Figure S4

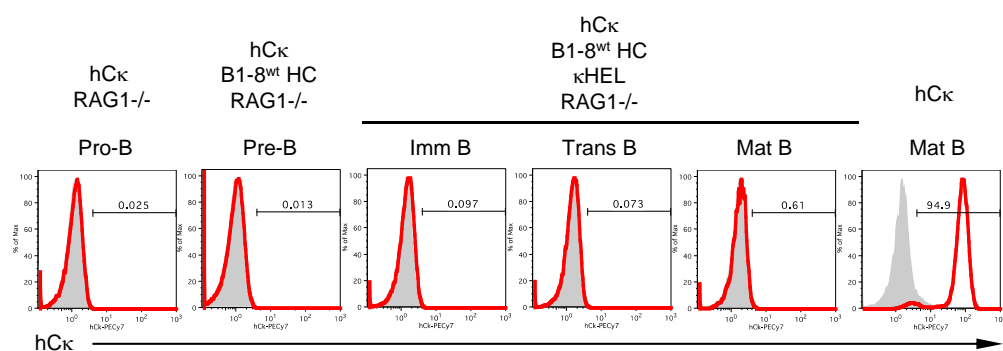

Supplement: S4 Fig — hCκ mice were crossed with B1-8wt HC/κHEL mice and back-crossed onto a RAG−/− background to obtain the depicted genotypes. Bone marrow cells were stained for surface markers and then fixed and permeabilized to analyze human Cκ expression by flow cytometry. Pro-B and pre-B cells are gated B220+ IgM−, immature (imm) B cells are gated B220+ IgM+ IgD−, transitional (trans) B cells are gated B220+ IgM+ IgDlow, and mature (mat) B cells are gated B220+ IgM+ IgDhigh. Mature B cells from a regular hCκ mouse served as a positive control. Grey shaded histograms show cells from a hCκ-negative control mouse. Results are representative of at least two independent experiments. (PDF) [file pone.0113824.s004.pdf]
